# Altered Induction of Reactive Oxygen Species by X-rays in Hematopoietic Cells of C57BL/6-Tg (CAG-EGFP) Mice

**DOI:** 10.3390/ijms22136929

**Published:** 2021-06-28

**Authors:** Cuihua Liu, Hirokazu Hirakawa, Takanori Katsube, Yaqun Fang, Kaoru Tanaka, Mitsuru Nenoi, Akira Fujimori, Bing Wang

**Affiliations:** 1 Molecular and Cellular Radiation Biology Group, Department of Charged Particle Therapy Research, Institute for Quantum Medical Science, Quantum Life and Medical Science Directorate, National Institutes for Quantum and Radiological Science and Technology, Chiba 263-8555, Japan; liu.cuihua@qst.go.jp (C.L.); hirakawa.hirokazu@qst.go.jp (H.H.); fang.yaqun@qst.go.jp (Y.F.); 2 Dietary Effects Research Group, Department of Radiation Effects Research, National Institute of Radiological Sciences, Quantum Life and Medical Science Directorate, National Institutes for Quantum and Radiological Science and Technology, Chiba 263-8555, Japan; katsube.takanori@qst.go.jp (T.K.); tanaka.kaoru@qst.go.jp (K.T.); 3 Human Resources Development Center, Quantum Life and Medical Science Directorate, National Institutes for Quantum and Radiological Science and Technology, Chiba 263-8555, Japan; nenoi.mitsuru@qst.go.jp

**Keywords:** green fluorescent protein (GFP), reactive oxygen species (ROS), ionizing radiation, hematopoietic cells, GFP transgenic mice

## Abstract

Previous work pointed to a critical role of excessive production of reactive oxygen species (ROS) in increased radiation hematopoietic death in GFP mice. Meanwhile, enhanced antioxidant capability was not demonstrated in the mouse model of radio-induced adaptive response (RAR) using rescue of radiation hematopoietic death as the endpoint. ROS induction by ex vivo X-irradiation at a dose ranging from 0.1 to 7.5 Gy in the nucleated bone marrow cells was comparatively studied using GFP and wild type (WT) mice. ROS induction was also investigated in the cells collected from mice receiving a priming dose (0.5 Gy) efficient for RAR induction in WT mice. Significantly elevated background and increased induction of ROS in the cells from GFP mice were observed compared to those from WT mice. Markedly lower background and decreased induction of ROS were observed in the cells collected from WT mice but not GFP mice, both receiving the priming dose. GFP overexpression could alter background and induction of ROS by X-irradiation in hematopoietic cells. The results provide a reasonable explanation to the previous study on the fate of cells and mice after X-irradiation and confirm enhanced antioxidant capability in RAR. Investigations involving GFP overexpression should be carefully interpreted.

## 1. Introduction

Application of reporter proteins is an indispensable tool in the research field of life sciences. Green fluorescent protein (GFP) of jellyfish is an unusual protein with visible absorbance and fluorescence, and GFP fluorescence emerges in the absence of substrates or cofactors because GFP self-contains a fluorescent p-hydroxybenzylidene-imidazolidinone chromophore in the peptide chains. As a unique bioindicator or biomarker, GFP is the first and the most used fluorescent protein in a variety of biosystems. It has become an important tool for measuring spatial and temporal patterns of gene expression, localization of proteins and cell tracking in living organisms [1,2,3]. The sensitivity of wild type GFP is below that of standard reporter proteins that utilize enzymatic amplification. Enhanced GFP (EGFP), achieved by human codon optimization and fluorophore mutation for increased fluorescence yield and improved expression in mammalian systems [4], is the most widely used in vivo protein marker, allowing observation of dynamic developmental processes in real time. It has made a significant contribution to the study of a number of different molecular processes during development and resulted in numerous promising discoveries [5]. In radiation biology investigations using mammalian cells, GFP is used to visualize modification of gene expression, signal transduction, cell metabolism, cell cycle change and cell death, providing critical information on the cellular response to ionizing radiation (IR). For example, cells expressing GFP were used to assess gene expression in response to UVC in space, signal pathway changes to accelerated heavy ions in a model of space environmental radiation conditions, cell cycle progression induced by an X-ray microbeam and killing efficacy by UV light of cancer cells [6,7,8,9]. GFP transgenic medaka fish were used to study responses of embryonic germ cells to gamma rays and of the thymus to X-rays and Fe-heavy ions [10,11]. In addition, GFP technologies were also applied to some experimental biosystems using lower organisms to evaluate radiation-responsive promoters and biological effects of chronic low-dose beta radiation from tritiated water [12,13]. GFP has revolutionized biological studies and made groundbreaking scientific achievements [14].

On the other hand, increasing evidence has shown alterations in biological properties and physiological functions of the cells and animals overexpressing transgenic GFP. Although GFP was believed to be biologically inert and even without noticeable adverse effects in vivo [15,16,17], more findings demonstrated the existence of abnormalities in cells and animals and that expression of EGFP in cells is not innocuous [18]. For example, in yeast and mammalian cell lines, GFP expression triggered changes in protein burden, proteome, myopathy, mitochondrial transcript expression and apoptosis [19,20,21,22,23]; in zebra fish, overexpressing GFP caused embryonic cardiac malfunction and defects in aerobic performance in adults [24], and in mice, expression of transgenic GFP resulted in dilated cardiomyopathy, earlier death and altered organ functions [25,26]. Of note, compared to their wild type (WT) counterparts, GFP transgenic cells and mice showed an altered response to insults including IR. For example, cells transduction of EGFP into human neuroblastoma cell lines markedly sensitized the cells and enhanced anticancer drug cytotoxicity [27]. Expression of GFP enhanced sensitivity to cytotoxic drugs and significantly changed transcriptional regulation of the mitochondrial genes in response to gamma irradiation [20,28]. In GFP transgenic mice, an altered response to total-body X-irradiation, from differential gene expression in hematopoietic cells to mouse killing, was demonstrated [29].

Reactive oxygen species (ROS) constitute a group of short-lived and highly reactive chemical molecules containing free radicals and peroxygen compounds. As critical signaling molecules, ROS play principal roles in the maintenance of normal physiological functions and homeostasis. However, excessive ROS could provoke damage to the redox balance and promote the oxidation of DNA bases, which can overload base excision repair pathways and thus increase the potential generation of double-strand breaks, cause damage to mitochondrial and nuclear DNA, proteins and lipids that link to a wide variety of pathologies and result in cell death and various health consequences [30,31,32,33,34,35,36,37]. ROS are generated during mitochondrial respiration and under various environmental stresses. Exposure to IR leads to oxidizing events that alter atomic structure through both direct interaction of radiation with target macromolecules and indirect interaction (namely, generation of ROS via products of water radiolysis) and cause DNA and subcellular organelle damage. Continuous activation and increase in endogenous and exogenous ROS could destroy the antioxidant system and stimulate production of more ROS, forming a cascade of amplified inflammatory responses, leading ultimately to cell death and tissue injury with both short- and long-term detrimental effects [32,38]. For conversion of immature EGFP to the fluorescent form and the maturation of EGFP, one equivalent of hydrogen peroxide (H_2_O_2_) per molecule of chromophore was produced [39,40]. It was believed that generation of H_2_O_2_ was at nontoxic levels [39], however, based on the results obtained in cell-free assays with GFP concentrations comparable to those in cells, Ganini et al., (2017) [41] first successfully showed increased production of extracellular H_2_O_2_ in HeLa cells stably expressing GFP. They further confirmed that many biological pathways were altered, particularly the pathways implicated in the pathophysiology of many diseases associated with oxidative stress, for example, genes activated by superoxide and hydrogen peroxide were upregulated in *E. coli* with EGFP expression, and GFP overexpression caused upregulation of several genes associated with inflammation in HeLa cells. Many studies also showed that redox signaling mechanisms induced by increased ROS (i.e., superoxide and H_2_O_2_) could result in altered gene expression of cell regulatory proteins affecting cell fate (proliferation, differentiation and death) [42,43,44,45], and cytotoxicity in cells and tissue abnormalities in animals overexpressing GFP could be explained well by increased ROS [18,19,25,26,27]. These results provided reliable evidence in bacterial and mammalian cells in the in vitro systems that enhanced ROS formation and alterations in oxidative stress genes in response to GFP expression in cells undergoing synthesis and maturation of GFP [46]. In our previous study using GFP transgenic C57BL/6-Tg (CAG-EGFP) mice, altered responses to total body irradiation (TBI) were first demonstrated at molecular, cellular and whole-body levels, such as differential gene expression and elevated apoptosis induction in hemopoietic cells and increased bone marrow lethality [29].

Radiation-induced adaptive response (RAR) is described as a phenomenon in which exposure to priming IR at a low dose could result in increased resistance to the challenge of IR at higher doses [47,48]. RAR has been observed in many in vitro, in vivo and ex vivo biosystems using a variety of cell and tissue types, biological endpoints and radiation qualities [49]. RAR has important implications for precision multimodality cancer treatment [50]. The probable underlying mechanisms involved in RAR are the transcription of many genes and the activation of numerous signaling pathways that trigger cell defenses which manifest as enhanced DNA repair, detoxification of free radicals and antioxidant production [51]. As a regulator of RAR, radiation-induced oxidative stress and its molecular downstream signaling pathways have a great impact on the induction of RAR. Radiation-induced oxidative stress could induce various molecular adaptors connected to RAR in the exposed cells, involve into proliferative responses, activate the intrinsic apoptotic pathway, cause inflammation and promote genetic instability [52]. Our previous work also demonstrated that RAR was diminished in GFP transgenic mice [29].

As the cytotoxic effects of IR are resulted from radiation-induced oxidizing events, the change of ROS is expected to play a critical role in response to IR in GFP transgenic mice, leading to an additive or even synergistic effect on the IR-induced detrimental effects. In the present work, both the background level of ROS and the induction of ROS by X-rays were comparatively studied in hematopoietic cells measured as nucleated bone marrow cells collected from the same strain GFP transgenic mice and their C57BL/6N wild type counterparts. The obtained results provide solid evidence showing an elevated ROS background and significantly enhanced ROS formation by IR in hematopoietic cells in GFP transgenic mice compared to those of their WT counterparts. These findings indicate that transgenic GFP expression in mammalian cells is not innoxious and suggest that GFP could alter cell phenotype and response to environmental insults such as IR, thus behaving as a confounder that affects the interpretation of experimental data obtained in biosystems using transgenic expression of GFP.

## 2. Results

### 2.1. General Physiological Conditions of the Mice

Both GFP mice and WT mice without morphological and behavioral abnormalities were used in the present study. As shown in Table 1, the mean body weight of the GFP mice was slightly lower at six postnatal weeks and markedly lower at eight postnatal weeks compared to that of their counterpart WT mice. No difference was found between the two groups of mice for the mean number of hematopoietic cells measured as nucleated bone marrow cells collected from both femurs of the mouse at postnatal eight weeks. According to our previous investigation, when compared to the WT mice, both the percentage of apoptotic cells and the expression level of proapoptotic gene Bax were slightly higher in nucleated bone marrow cells of the GFP mice, while the expression level of antiapoptotic gene Bcl-2 was slightly lower [29].

Collectively, these results clearly showed that although GFP mice were reported as normal and healthy [16], and GFP mice without morphological and behavioral abnormalities were used in our investigations, alteration in physiological conditions was demonstrated at molecular, cell and whole-body levels when compared to their counterpart WT mice.

### 2.2. Induction of ROS by X-rays in the Hematopoietic Cells

**Figure 1:**
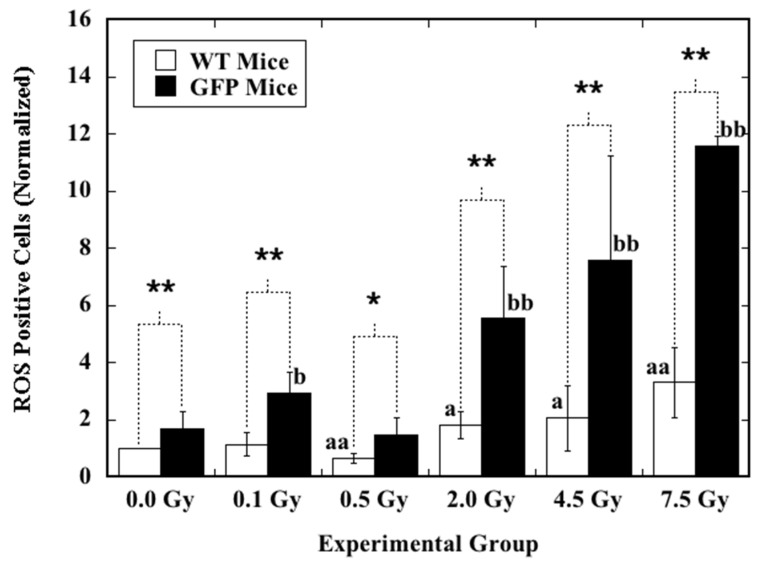
Induction of ROS by ex vivo X-irradiation in hematopoietic cells from mice at eight postnatal weeks. Nucleated bone marrow cells were irradiated (0.1–7.5 Gy) or sham-irradiated (0.0 Gy) with X-rays, and induction of ROS was measured. Data are presented as ratios normalized to the ROS results of the cells from sham-irradiated control WT mice. One (*) and two asterisks (**) indicate statistically significant differences (* *p* < 0.05; ** *p* < 0.01) between the two groups that were compared. For comparison of the irradiated group to the sham-irradiated control in WT mice, one letter “a” and two letters “aa” stand for statistically significant differences at *p* < 0.05 and *p* < 0.01, respectively. For comparison of the irradiated group to the sham-irradiated control in GFP mice, one letter “b” and two letters “bb” stand for statistically significant differences at *p* < 0.05 and *p* < 0.01, respectively.

The background level of ROS and induction of ROS by ex vivo X-irradiation in the hematopoietic cells measured as nucleated bone marrow cells collected from the femurs of mice at eight postnatal weeks were analyzed by flow cytometry (Figure 1). For the background level of ROS in the cells from the sham-irradiated mice (0.0 Gy), a significantly higher proportion of ROS-positive cells was observed in GFP mice (1.68 times) when compared to that in the WT mice. The value of ROS-positive cells was 2.60 ± 1.01% and 1.55 ± 0.23% in GFP mice and WT mice, respectively. After exposure to X-irradiation, in each irradiated group receiving the same dose, a statistically marked increase in induced ROS was always observed in the cells from GFP mice when compared to those from WT mice. Except for the 0.5 Gy irradiated group, X-irradiation induced a dose-dependent increase in ROS in the cells from both GFP mice and WT mice. After exposure to 0.5 Gy, when compared to the sham-irradiated control, the ROS level was significantly reduced in the cells from WT mice, while no statistically detectable difference was observed in the cells from GFP mice.

These results showed clearly that in the hematopoietic cells of GFP mice, the background level of ROS was significantly elevated. The cells were much more sensitive to X-irradiation, measured as increased induction of ROS. The priming dose efficient for the induction of RAR in the WT mice did not result in reduced induction of ROS in the cells from GFP mice.

### 2.3. Effect of Priming TBI on Induction of ROS by Ex Vivo X-Irradiation in the Hematopoietic Cells

**Figure 2:**
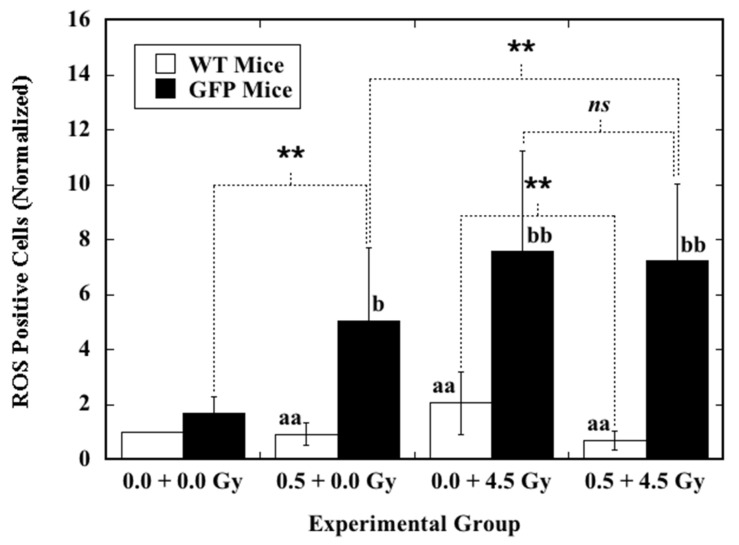
The effect of priming TBI at six postnatal weeks on ROS induction by ex vivo X-irradiation in the hematopoietic cells from mice at eight postnatal weeks. The animals were irradiated with a priming dose of 0.5 Gy or sham-irradiated (0.0 Gy) at six postnatal weeks. Nucleated bone marrow cells collected from the animals at eight postnatal weeks were irradiated (4.5 Gy) or sham-irradiated (0.0 Gy) with X-rays, and induction of ROS was measured. “0.0 + 0.0 Gy” represents the cells from mice receiving sham-irradiation at six postnatal weeks and being sham-irradiated ex vivo. “0.5 + 0.0 Gy” represents the cells from mice receiving a priming dose of 0.5 Gy at six postnatal weeks and being sham-irradiated ex vivo. “0.0 + 4.5 Gy” represents the cells from mice receiving sham-irradiation at six postnatal weeks and being irradiated with 4.5 Gy ex vivo. “0.5 + 4.5 Gy” represents the cells from mice receiving a priming dose of 0.5 Gy at six postnatal weeks and being irradiated with 4.5 Gy ex vivo. Data are presented as ratios normalized to the ROS results of the cells from WT mice receiving sham-irradiation at six postnatal weeks and being sham-irradiated ex vivo. Two asterisks (**) indicate a statistically significant difference (** *p* < 0.01) between the two groups that were compared. Letters “*ns*” indicate no statistically significant difference between the two groups that were compared. For comparison of the irradiated group to the sham-irradiated control in WT mice, two letters “aa” stand for statistically significant differences at *p* < 0.01. For comparison of the irradiated group to the sham-irradiated control in GFP mice, one letter “b” and two letters “bb” stand for statistically significant differences at *p* < 0.05 and *p* < 0.01, respectively.

The effect of priming TBI with 0.5 Gy at six postnatal weeks on the level of ROS and induction of ROS by ex vivo X-irradiation in the nucleated bone marrow cells collected from the femurs of mice at eight postnatal weeks was analyzed by flow cytometry (Figure 2). When compared to the background level of ROS in the cells from sham-irradiated mice (0.0 + 0.0 Gy), the level of ROS was significantly reduced in the cells collected from WT mice that received priming TBI (0.5 + 0.0 Gy). In contrast, the level of ROS was markedly elevated in the cells from GFP mice receiving the same treatment (0.5 + 0.0 Gy). After ex vivo X-irradiation with 4.5 Gy, induction of ROS in the cells from WT mice that received priming TBI (0.5 + 4.5 Gy) was significantly reduced compared to that in the cells from the WT mice that received sham priming TBI (0.0 + 4.5 Gy). On the other hand, this phenomenon was not observed in the cells from GFP mice with the same treatment. In addition, in cells from GFP mice, ex vivo X-irradiation always caused markedly increased ROS induction regardless of priming TBI. In cells from WT mice, priming TBI always induced significantly reduced ROS induction, regardless of ex vivo X-irradiation.

These results showed clearly that priming TBI of the animals could significantly reduce the ROS level in the hematopoietic cells of WT mice but markedly increase the ROS level in the cells of GFP mice. Furthermore, priming TBI of the animals could significantly reduce the ROS induction by ex vivo X-irradiation in the hematopoietic cells of WT mice, but it did not have such an impact on the cells of GFP mice that received the same treatment. These findings also confirm the positive correlation between induction of radio-resistance and increased antioxidant capability, which is one of the underlying mechanisms for RAR.

## 3. Discussion

ROS play central roles in regulating the main pathways of apoptosis, and proper regulation of apoptosis is essential for maintaining normal cellular homeostasis and normal physical functioning. Under normal physiological conditions, redox homeostasis is a consequence of the equilibrium between generation of ROS and functioning of the antioxidant system. At low to modest levels, ROS are essential for regulation of normal physiological functions such as gene expression, cell cycle progression and proliferation and cell death. ROS also play a critical role in the immune system and maintenance of the redox balance and activate various cellular signaling pathways as important regulators. On the other hand, excessive ROS levels could cause excessive redox stress, induce intra- and inter-mitochondrial redox-environment changes, leading to further ROS release through a mechanism called ROS-induced ROS release. These changes could result in longer mitochondrial permeability transition pore openings that may release a ROS burst, leading to destruction of mitochondria, damage to DNA, proteins, lipids, membranes and organelles, thus causing pathological elimination of mitochondria and activation of cell death processes and contributing to pathologic conditions, including triggering apoptosis, tumor promotion and progression [53]. Thus, ROS play indispensable roles in both cell signaling and regulation of the main apoptosis pathways that are mediated by mitochondria, death receptors and the endoplasmic reticulum [54,55]. Our previous study demonstrated that, compared their wild type counterparts, GFP mice had a slightly elevated level of apoptosis in the nucleated bone marrow cells, and after TBI, there was a significant increase in both apoptosis induction and mouse killing effect from hematopoietic death. The present work further confirmed that in GFP mice, these cells had a higher level of background ROS and after radiation exposure, induction of ROS was markedly increased. Of note, ROS as mediators play diverse roles in cell cycle regulation via incorporating phosphorylation, ubiquitination and receptor activation, involved in the integrity and survival of the cell [56,57,58,59,60]. Our previous work also exhibited the differential induction of cell cycle arrest in cells from GFP mice compared to that in their wild type counterparts [29]. Furthermore, the results on priming TBI-induced reduced ROS induction by ex vivo challenge X-irradiation in the hematopoietic cells of WT mice but not in the cells of GFP mice receiving the same treatment also confirm the positive correlation between induction of radio-resistance and increased antioxidant capability, which could explain, to a certain extent, the mechanisms underlying diminishment of RAR in GFP mice. Together with the findings in our previous work on proapoptotic gene induction, all these results were consistent with each other, pointing to the causative role of excessive ROS in the increased induction of hematopoietic death in GFP mice.

According to Ansari et al. (2016), in GFP transgenic animals, cellular damage occurs possibly due to direct injury by ROS generation, initiation of apoptosis and damage by immune mechanisms [18]. As a matter of fact, existence of abnormalities in terms of cytotoxicity, immunogenicity and overall function was reported in cells and animals expressing transgenic GFP [18]. In addition to GFP resulting in oxidative stress, GFP-induced immunologic responses could be another important contributing factor responsible for the altered response of GFP mice to IR. As a matter of fact, many studies show that introduction and expression of GFP could induce immunologic responses in vivo in mice, monkeys and humans. Although the literature on GFP cellular metabolism and GFP molecular interactions within the cell is still in its infancy and some early investigations did not report noticeable alterations, accumulating new evidence from later investigations, particularly phenotype studies, points to a clear association of GFP expression with alterations in phenotype, toxicity and responses to exogenous insults at cellular, tissue and whole-body levels. For example, early morphological investigations reported that GFP expression did not induce loss of viability or confer growth disadvantage in cultured plant cells and established mammalian cell lines [61,62], transfection with plasmid DNA encoding GFP did not affect differentiation and function of mouse neuronal progenitors [63,64] and no noticeable developmental anomalies were observed in GFP transgenic flies and mice [16,61,65,66]. However, later studies showed that GFP transgenic mice developed moderate to severe dilated cardiomyopathy [25] due to increased activity of calmodulin-dependent protein kinase II by EGFP overexpression, which disrupted normal cellular signaling [67]. Phenotype studies also demonstrated GFP-associated changes in cell and animal models. Expression of GFP caused toxicity in mammalian cell lines underlying the mechanisms from free radical-associated phototoxicity to both the excitation and undefined cellular effect of GFP [19]. Especially in the in vivo studies, one of the most concerning issues is the exogenous introduction of the GFP gene from nonmammalian jellyfish into mammalian cells in vivo augmenting the immune response to the novel protein product GFP. The processed peptides derived from GFP and presented by the major histocompatibility complex on the cell surface induce immunogenicity that manifests as cytotoxic T lymphocyte (CTL) immune responses against cells expressing GFP. In line with this, to date, immunological rejection due to exogenous introduction of the GFP gene has been reported in monkeys that underwent nonmyeloablative irradiation, showing antibody and CTL responses against EGFP-expressing CD34+ bone marrow cells and their progeny and loss of these genetically modified cells in peripheral blood [68]. Development of CTL responses specific to GFP eliminated GFP-expressing cells in humans [69]. GFP expression in dendritic cells enhanced their immunogenicity and elicited specific CTL responses in humans [70]. Furthermore, CTL responses to GFP were reported in multiple mouse models. For example, GFP expression in breast cancer cells induced proteome modifications, manifesting as changes in expression of proteins associated with protein folding, cytoskeletal organization and cellular immune response [26]. In transplantable mouse models, leukemia and lymphoma cells expressing high levels of EGFP showed a drastic decrease in disease development when transplanted into immunocompetent mice due to development of high CTL responses [71,72,73]. Immune stimulation against lymphoma cells expressing high levels of EGFP could also be induced by immunization with transduced dendritic cells expressing EGFP [74]. On the other hand, as GFP transgenic mice are immunologically tolerant to GFP, it would be mechanistically different from the studies mentioned earlier on the administration of GFP-expressing cells to immunocompetent recipients inducing an immune response. The immunogenic response induced by ROS in GFP transgenic mice would be through inflammatory and autoimmune responses. Transgenic mice that express nonfunctional mutant GFP (nonfluorescent EGFP) are immunologically tolerant to the cells expressing the active forms of GFP [75]. However, in the GFP transgenic mice used in the present work, EGFP was fluorescent and expressed in almost all the tissues except erythrocytes and hair [16]. As a matter of fact, several in vivo studies claimed that GFP could impair transgenic animals’ health. Of note, in a newly published review, Lipták et al., (2019) summarized the health consequences in these most popularly used GFP transgenic mice, including growth retardation, mild glomerulosclerosis and proteinuria and neuropathology due to ROS accumulation and ROS-induced inflammatory response [76]. Prolonged and uncontrolled ROS production and accumulation could induce inflammation and tissue damage, leading to apoptosis and autoantigen structural changes that result in novel specificities [77,78]. It was shown that ROS are implicated in the pathogenesis of autoimmune diseases, not only in the initiation of the autoimmune response but also in its amplification and spreading to novel epitopes through the unmasking of cryptic determinants [77]. Taken together, there is a high possibility that accumulation of ROS due to transgenic introduction and expression of the GFP gene could consequently augment and activate pathological immune responses in the GFP transgenic mice used in our previous and current investigations, thus playing a causative role in the altered responses of GFP mice to IR. Further studies are needed to verify the immune conditions of the animals and clarify the effect of the GFP-induced immune response.

Elevated background and enhanced induction of ROS by X-irradiation demonstrated in the nucleated bone marrow cells from GFP mice further confirmed that the use of GFP with the presumption of its biological inactivity was invalid. In fact, GFP transgenic cells also showed increased sensitivity to other insults such as drugs [27,28]. In the present work, we also found that the nucleated bone marrow cells from GFP mice were much more responsive to cold stress, an inducer known to trigger ROS production in cells [79,80]. After keeping the cells on ice for 3 h, the percentage of ROS-positive cells increased significantly more (*p* < 0.01) among the cells of GFP mice (from 2.60 ± 1.01% to 14.04 ± 1.90%) than among those of WT mice (from 1.55 ± 0.23% to 4.36 ± 1.73%). Collectively, these findings indicate that GFP transgenic mice are not as “normal” as their wild type counterparts. There could be major influence on the interpretation of the results obtained in studies using GFP transgenic mice. These findings also suggest that it is critical that investigations using GFP techniques for cell labeling and in vivo cell tracing receive critical validation with alternative methodologies and the results are carefully interpreted. On the other hand, from a different point of view, GFP transgenic mice could also provide a model to investigate the underlying mechanisms of alteration in physiology and responses to insults such as IR in GFP transgenic animals. Further mechanistic studies are warranted. For example, what are the exact mechanisms underlying the elevated endogenous ROS generation in relation to the production of transgenic GFP? What is the interplay between elevated ROS background, enhanced ROS induction by IR, alteration in proapoptotic gene expressions, change of cycle arrest, elevation in apoptosis induction, diminished induction of RAR and increased sensitivity to IR-induced bone marrow death in GFP transgenic mice? These questions should be carefully verified in future investigations.

## 4. Materials and Methods

### 4.1. Animals

Both C57BL/6-Tg (CAG-EGFP) female mice and C57BL/6N wild type female mice at five or seven postnatal weeks were purchased from SLC, Inc. (Hamamatsu, Japan). The C57BL/6-Tg (CAG-EGFP) mice were originally produced by Okabe et al. (1997) and belonged to the “green mice” line 131 [16]. In the mouse genome, the transgene integration chromosomal locus was on chromosome 14 D1 [81]. The enhanced green fluorescent protein (EGFP) was expressed by the CAG promoter (pCAGGS-EGFP), and almost all tissues and cells (except erythrocytes and hair) of the mice fluoresced bright green [16,82]. The homozygous animals that showed no abnormal physical appearance and behavioral abnormalities were used in the present work. In this paper, the short term “GFP mice” was used to denote “C57BL/6-Tg (CAG-EGFP) mice”, and “WT mice” was used to represent “C57BL/6N wild type mice.” The WT mice were used as counterparts of the GFP mice. To avoid possible effects from the developmental condition of the animals, any mouse with a significantly different body weight, namely more or less than the mean ± 2 standard deviations (SD) of all the animals upon arrival, was omitted from this study. From both GFP and WT mice, the selected animals were randomly assigned to 2 experimental groups either as the sham-irradiated group or the irradiated group. All animals were maintained in a conventional animal facility under a 12 h light–12 h dark photoperiod, controlled temperature (23 ± 2 °C) and humidity (50 ± 10%), housed in autoclaved aluminum cages (3 mice per cage) with sterilized wood chips and allowed access to a standard laboratory chow MB-1 (Funabashi Farm Co., Funabashi, Japan) and acidified water (pH = 3.0 ± 0.2) ad libitum.

Based on preliminary trials, three mice were used for each experimental datum point in the present work. The experiment was repeated at least once for the high-dose (2.0, 4.5 and 7.5 Gy) irradiated groups and twice for the low-dose (0.1 and 0.5 Gy) irradiated groups. In each experiment using either WT mice or GFP mice, three animals were used in the nonirradiated (0.0 Gy) group. The data presented in this paper were obtained using 18 mice in the nonirradiated group, 9 mice in each of the low-dose irradiated groups, and 6 mice in each of the high-dose irradiated groups. All experimental protocols (Experimental Animal Research Plan No. 16-2010-5 on 12 July 2019 and No. 11-1003-5 on 7 March 2016, and Research Plan Using Genetically Modified Organisms No. H25-3-3 on 20 March 2018) involving mice were reviewed and approved by The Institutional Animal Care and Use Committee of the National Institute of Radiological Sciences, Quantum Life and Medical Science Directorate, National Institutes for Quantum and Radiological Science and Technology, Japan. The experiments were performed in strict accordance with the Institutional Guidelines for the Care and Use of Laboratory Animals.

### 4.2. Collection of the Nucleated Bone Marrow Cells

The nucleated bone marrow cells were collected according to [29]. In brief, mice at eight postnatal weeks were euthanized by CO_2_ asphyxiation. Both femurs of each mouse were removed, and the bone marrow tissues were collected by flushing femurs with phosphate-buffered saline free from calcium and magnesium ions. After treating bone marrow tissues with tris-buffered ammonium chloride for the lysis of erythrocytes and washing with RPMI medium 1640 (Cat. 06261-65, with L-glutamine and without phenol red, Nacalai Tesque, Inc., Tokyo, Japan), single cell suspensions of dissociated nucleated bone marrow cells were filtered through a 40 μm cell strainer (Corning, Inc., New York, NY, USA) and then counted for further use.

### 4.3. Irradiation

X-rays were generated with an X-ray machine (TITAN-E320, Shimadzu, Industrial Systems Co., Ltd., Otsu, Japan) operated at 200 kVp and 20 mA, using a 0.50 mm Al + 0.50 mm Cu filter. An exposure rate meter (AE-1321 M, Applied Engineering Inc., Tokyo, Japan) with an ionization chamber (C-110, 0.6 mL, JARP, Applied Engineering Inc., Tokyo, Japan) was used for the dosimetry. For irradiation of the 6-weeks-old mice, animals were held in an acryl container and exposed to total body irradiation (TBI) at a dose of 0.5 Gy with a dose rate at 0.3 Gy/min without anesthesia at room temperature. The dose of 0.5 Gy of TBI is a priming dose efficient for the induction of RAR, measured as reduced induction of radiation-induced hematopoietic death in WT mice but not in GFP mice [29]. For ex vivo irradiation of the nucleated bone marrow cells, cell suspensions in lightproof brown tubes (Cat. 616283, Greiner Bio-One International GmbH, Kremsmünster, Austria) were irradiated at a dose ranging from 0.1 to 7.5 Gy or sham-irradiated (0.0 Gy) at room temperature. Two high doses (4.5 Gy and 7.5 Gy) were used based on previous work [29]. A dose of 4.5 Gy was sufficient to induce differential effects on induction of gene expression, cell cycle change and apoptosis in the nucleated bone marrow cells from WT mice and GFP mice. A dose of 7.5 Gy caused, respectively, 94% and 100% lethality in female WT mice and GFP mice in the 30-day survival test. The dose rate was about 0.2, 0.4 and 1.5 Gy/min, respectively, for the delivery of a dose from 0.1, 0.5 and 2.0 to 7.5 Gy.

### 4.4. Flow Cytometric Analysis of ROS

**Figure 3:**
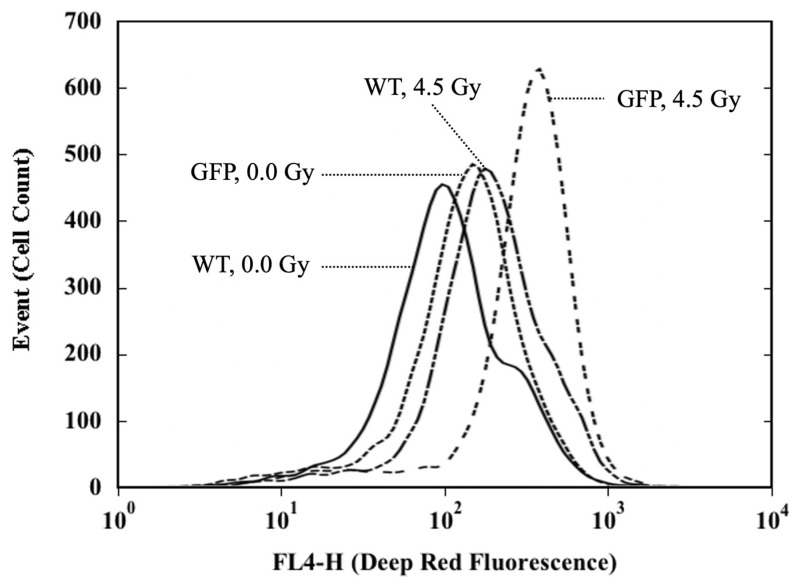
Modal representation of the number of cells as a function of the fluorescence emission intensity of ROS detector Deep Red. Nucleated bone marrow cells collected from animals at eight postnatal weeks were ex vivo irradiated (4.5 Gy) or sham-irradiated (0.0 Gy) with X-rays, and induction of ROS was measured flow cytometrically using channel FL-4. The fluorescence signal was monitored at an excitation wavelength of 650 nm and an emission wavelength of 675 nm for Deep Red detection. “0.0 Gy” represents cells receiving sham-irradiation. “4.5 Gy” represents cells receiving 4.5 Gy.

For the analysis of the background level and induction of ROS by X-rays in the nucleated bone marrow cells, the Cellular ROS Assay Kit (Deep Red) (ab186029, Abcam, Cambridge, UK) was used according to the manufacturer’s instructions with some modifications [38,83]. The kit provides a sensitive fluorometric one-step assay to detect intracellular ROS (especially superoxide and hydroxyl radical) in live cells. In brief, each sample containing 2.5 × 10^5^ cells in 200 μL RPMI medium supplemented with 10% heat-inactivated fetal bovine serum was cocultured with ROS Deep Red dye for 30 min at 37 °C in a 5% CO_2_ incubator. After X-irradiation, the cultures were incubated for another 30 min, fixed with 4% paraformaldehyde phosphate buffer solution (Cat. 09154-85, Nacalai Tesque, Inc., Tokyo, Japan) at a final concentration of about 1.0% and then subjected to flow cytometric analysis. Mean fluorescence intensity was measured with a FACSverse^TM^ flow cytometer (Becton-Dickinson Biosciences, Franklin Lakes, NJ, USA) using channel FL-4, and the fluorescence signal was monitored at an excitation wavelength of 650 nm and an emission wavelength of 675 nm for ROS Deep Red detection (Figure 3). A total of 15,000–30,000 live cells were analyzed in each sample. The data were analyzed using Becton Dickinson FACSuite™ Software (Version 1.0.6, Becton-Dickinson Biosciences, San Jose, CA, USA). Results are expressed as the percentage of ROS-positive cells in nucleated bone marrow cells. Data are presented as ratios (mean ± SD) normalized to the ROS results of the sham-irradiated cells from 8-weeks-old WT mice.

All experiments involving manipulation of bone marrow cells and use of the Cellular ROS Assay Kit were conducted under dark if possible to avoid the effect of lights.

### 4.5. Statistical Analysis

Statistical evaluation of the data was done with Student’s *t*-test for the difference between two groups. Statistical significance was assigned to *p* < 0.05.

## 5. Conclusions

Results obtained in the present work further provide solid evidence that shows (1) elevated ROS background and significantly enhanced ROS formation by IR in the hematopoietic cells of GFP transgenic mice compared to those of their WT counterparts and (2) priming TBI significantly reducing both the ROS level and the challenge IR-induced ROS in the hematopoietic cells from WT mice but not in the cells from GFP mice. These findings indicate that GFP expression by transgenic introduction of the GFP gene from nonmammalian jellyfish into the mouse genome is not innoxious. These findings also collaterally confirm the positive correlation between induction of radio-resistance and increased antioxidant capability. Given the interference with redox measurements in the cell, overexpression of GFP could alter the cell phenotype and response to environmental insults such as IR. Of special note, results obtained from experiments involving GFP expression should be carefully interpreted and further validated using alternative methodologies.

## Figures and Tables

**Table 1:** Body weight and number of hematopoietic cells.

Mice	Body Weight (g) at 6 Postnatal Weeks	Body Weight (g) at 8 Postnatal Weeks	Body Weight (g) at 8 Postnatal Weeks (with 0.5 Gy TBI at 6 Postnatal Weeks)
WT	16.7 ± 0.6	18.4 ± 0.5	18.7 ± 1.0
GFP	16.5 ± 0.7	17.8 ± 0.5 *	16.4 ± 0.6 *^,#^

* Statistically significant difference at *p* < 0.05 between the body weight of WT mice and GFP mice at eight postnatal weeks. ^#^ Statistically significant difference at *p* < 0.05 between the body weight of the group receiving priming TBI and the group without priming TBI in GFP mice at eight postnatal weeks.

## Data Availability

Data supporting the findings of the present study are available within the article. Raw data are available from the authors (C.L. and B.W.) upon reasonable request.
